# Baclofen Induced Encephalopathy in a 6-Year-Old Boy with Advanced Renal Failure

**Published:** 2015

**Authors:** Majid Malak, Mohammad Barzegar

**Affiliations:** 1 Pediatric Nephrologist, Pediatric Health Reseaech Center, Tabriz University of Medical Science, Tabriz, Iran

**Keywords:** Baclofen, Children, Renal, Encephalopathy

## Abstract

Baclofen is a drug for many diseases for all ages, but it is hazardous in patients with renal failure. This article talks about a case of baclofen overdose in a child with renal failure. A 6-year-old boy admitted to the emergency department with a loss of consciousness, hypotonia, and areflexia following administration of 20 mg baclofen (1mg/kg/daily) in total dose for his voiding dysfunction. His laboratory tests showed advanced renal failure. After withholding the medication and supportive therapy, he recovered completely after two days. After arousal, he complained of insomnia, strange sensations on the skin, intentional tremors, and ataxia. He left the hospital in good condition in three days. Renal function control before baclofen administration is mandatory especially in high-risk groups. A total dose of 1mg/kg lead to encephalopathy in children with advanced renal failure, with subtle persistent complaints persist are often overlooked for a while.

## Introduction

Baclofen or 4-amino-3 parachlorophenol gamma amino butyric acid (GABA) as a GABA receptor agonist is used widely in neurologic and spasmodic conditions such as cerebral palsy (CP). Baclofen is capable of inhibiting monosynaptic and polysynaptic reflexes at the spinal level possibly by hyperpolarization of afferent terminals (1). Some studies consider it a safe medication (2-4), while other reports indicate that baclofen may be associated with widespread problems in cognition, movement, and cardiorespiratory fields especially as regards renal failure; a higher age, and some grade of neurological problems (5). In this case report, we review the clinical findings of a child who was affected to baclofen neurotoxicity shortly after its use.

## Case History

A 6-year-old boy with voiding dysfunction was admitted to the emergency department due to loss of consciousness, loss of deep tendon reflex, and mydriasis. His drug history indicated that he had used baclofen two 10 mg doses of baclofen during the previous 12 hours. On his first visit, his Glasgow coma scale was 10, his blood pressure was 85/50, and hypotonia and loss deep tendon reflex were prominent. On day one, he could open his eyes for a while without orientation to place and time, had jerking movements, and muscle twisting while sleeping. On the second day, he recognized his parents, he had intention tremors and ataxia, and he had trouble falling in sleep, and indicated that he felt skin dysesthesia sensations (tactile hallucinations). . His laboratory tests were as follows: BUN 60 ( normal (8– 18 mg/dl) creatinine, 3.2 ( normal for age: 0.2–0.8 mg/ dl), sodium: 141 ( normal 135–155 meq/dl), potassium: 5 (normal 3–6 meq/dl), Blood sugar: 90 (normal: 47– 110mg/dl) and his EKG showed normal rate and rhythm with normal QTc 0.4 msec (normal< 0.45 msec). He left the hospital in good condition after 3 days.

## Discussion

Baclofen is a well-known medication that can be used successfully in immature newborn with hypertonia. Its starting dose is 0.5 mg/kg/d and can be increased weekly to a final dose of 1.5 mg/kg/d (6). There are also many reports of baclofen overdoses in healthy adults who used large doses for an extended period, e.g. for one month. They had hypotonia, bradycardia, hallucinations, respiratory depression with seizures, or movement disorders such as Huntington chorea (7-8). In advanced renal impairments, baclofen is associated with drug toxicity at doses as low as 15 mg. In another reported case, a 60-year-old uremic man affected with hiccups in spite of taking baclofen for 2-day or another 69-year-old man who used 20 mg. These cases show that baclofen toxicity was caused by renal failure (5). In spite of 5 mg recommended daily dose for patients with renal failure, nearly all reported cases were toxic by using a total dose of 15–30 mg in less than 4 days. They usually recovered within 3 days by withholding the drug while most of them were under hemodialysis or peritoneal dialysis (5,8). Our case was a boy with advanced renal failure without need to dialysis who lost consciousness and had 20 mg (1mg/kg) of baclofen in one day. In fact, our case shows that baclofen without regard to age or weight with total doses greater than 15 mg can be hazardous to patients with advanced renal failure with a rapidly developing toxicity in 24 hours. In our case, our case also recovered in 2 or 3 days after drug withholding without need for emergency dialysis like in other reports (5,9). Unconsciousness was his main symptom but like the other studies (7), our case showed hypotonic areflexia and some movement disorders such as intention tremor, ataxia, and muscle twisting, or a choreiform movement. Some psychiatric reactions may occur after long-term baclofen usage. Mainly by intrathecal form, although in a case who used oral form for one year had developed insomnia and auditory and visual hallucination after discontinuing baclofen (10). Our case showed subtle and more persistent complaints after just one day of baclofen overdose. He had insomnia after recovery and dysesthesia described as something moving on his hands that can be considered a tactile hallucination. In conclusion, Baclofen is a widely used drug that is prescribed in patients with renal failure for many purposes include voiding dysfunction or intractable hiccup. It is also associated with lethal side effects. Our case shows that a total dose of 15-20 mg can be addressed as a hazardous dose for children with advanced renal failure. It indicates similar toxic symptoms and recovery as other reports have indicated without the need for dialysis. 

## Author Contribution

Barzegar M : scientific consultant Majid Maleki : literature review and writing first draft of the manuscript
